# Retrospectively gated intra-cardiac 4D flow CMR using spiral k-space trajectories

**DOI:** 10.1186/1532-429X-15-S1-O64

**Published:** 2013-01-30

**Authors:** Sven Petersson, Andreas Sigfridsson, Carl Johan Carlhall, Tino Ebbers

**Affiliations:** 1 Linköping University, Linköping, Sweden; 2 Center for Medical Image Science and Visualization (CMIV), Linköping, Sweden; 3 Biomedical Engineering, ETH Zurich, Zurich, Switzerland

## Background

Time-resolved three-dimensional phase contrast CMR (4D flow) is a powerful tool for hemodynamic assessment in the cardiovascular system. However, long scan times have hindered the application of the method in many cases. By using spiral readout trajectories, improved efficiency provides a means of reducing scan times without decreasing SNR. Furthermore, spiral acquisition offers increased robustness in areas with accelerating flow. Spiral readouts have previously been used for rapid 4D flow measurements in the aorta using prospective gating [1]. Using retrospective gating, the entire cardiac cycle is covered, which allows analysis of late diastole and tracking of blood over a complete cardiac cycle. These are crucial for cardiac 4D flow studies, and allow for pathline based data quality assessment.

The aim of this work is to develop a retrospectively gated 4D flow sequence using a stack of spiral readouts for the measurement of intra-cardiac velocities.

## Methods

A retrospectively gated 4D flow sequence using a stack of spirals was implemented on a Philips Achieva 1.5T. The following parameters were used for the spiral measurement: field of view 180x180x112 mm^3^; voxel-size 2.8 mm isotropic; VENC 1.2 m/s; temporal resolution 48.8 ms; spiral duration 5.5 ms; 10 spiral interleaves. A Cartesian scan with the same spatial resolution and field of view using a SENSE reduction factor of 2 was carried out for comparison.

The data quality was evaluated by comparison with 2D through-plane velocity measurements in the proximal ascending aorta and quantitative pathline analysis. Pathlines were released backward and forward from the left ventricle (LV) to compute inflow and outflow volumes for the LV [2]; a small difference between the inflow and outflow values indicates good data quality. Furthermore, the outflow was compared to planimetry of CINE short axis images. To facilitate peak-flow comparison, the 4D flow data was reformatted to the same slice location as the 2D data.

## Results

**Figure 1:**
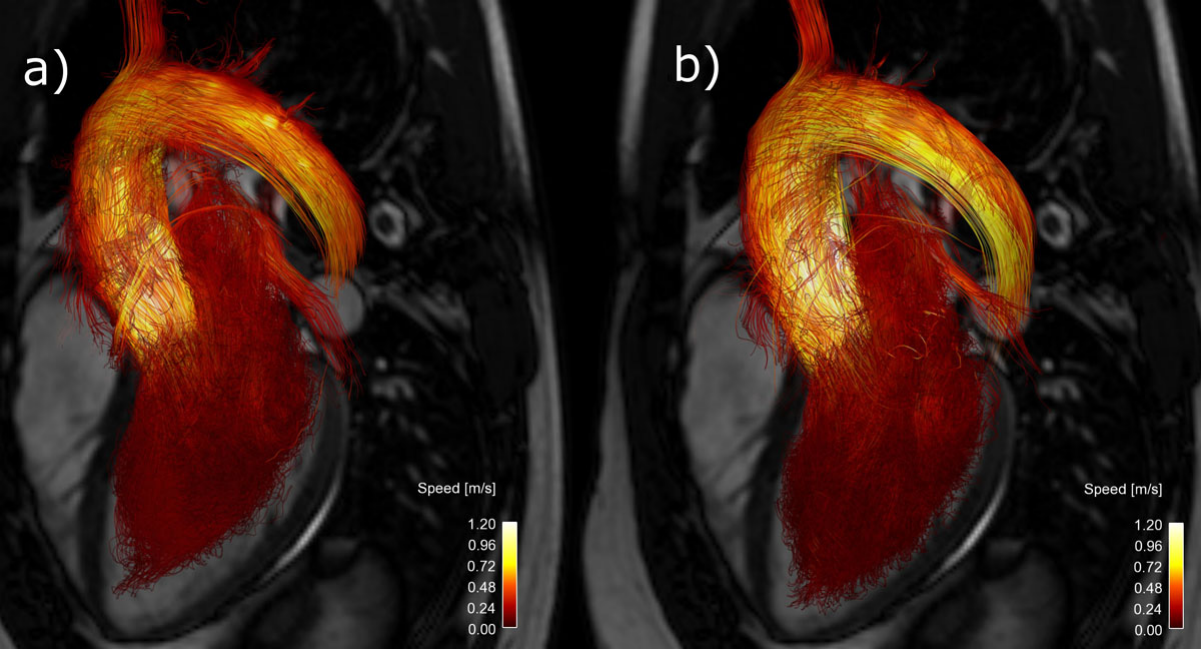
Pathlines visualizing the blood flow going from the left atrium, trough the ventricle, out in the aorta from 4D flow measurements using a) spiral readouts and b) cartesian readouts.

Using spiral k-space trajectories, a 62 % reduction in scan time could be achieved compared to the Cartesian scan, as shown in table 1. Quantitative pathline analysis and comparison with the 2D through-plane measurement does not show any decrease in data quality. Visual inspection of the pathlines did not reveal any major differences in data quality (see figure 1).

**Table 1:** Data quality assessment (one 32-year-old volunteer, heart rate 58)

	Inflow [ml]	Outflow [ml]	Peak flow [ml/s]	Scan time [min]	Scan time without nav [min]
Spiral	121*	119*	533	12	8:48
Cartesian	117*	118*	545	30	22:52
2D Aorta	N/A	128	613	N/A	N/A
Planimetry (Stroke volume)	N/A	124	N/A	N/A	N/A

* Inflow and outflow based on pathline analysis.

## Conclusions

A retrospectively gated spiral 4D flow sequence was successfully implemented. The spiral readouts resulted in more than a two-fold reduction in scan time compared to a conventional Cartesian scan, which was already accelerated using parallel imaging, without any notable loss in data quality. Additional improvements in scan time and resolution can be obtained by using parallel imaging. Further studies on healthy volunteers and patients will allow more detailed evaluation of the advantages of spiral 4D flow.

## Funding

Swedish Research Council.
